# Sex, Subdivision, and Domestic Dispersal of *Trypanosoma cruzi* Lineage I in Southern Ecuador

**DOI:** 10.1371/journal.pntd.0000915

**Published:** 2010-12-14

**Authors:** Sofía Ocaña-Mayorga, Martin S. Llewellyn, Jaime A. Costales, Michael A. Miles, Mario J. Grijalva

**Affiliations:** 1 Centro de Investigación en Enfermedades Infecciosas, Pontificia Universidad Católica del Ecuador, Quito, Ecuador; 2 London School of Hygiene and Tropical Medicine, London, United Kindom; 3 Biomedical Sciences Department, College of Osteopathic Medicine, Tropical Disease Institute, Ohio University, Athens, Ohio, United States of America; Institute of Tropical Medicine, Belgium

## Abstract

**Background:**

Molecular epidemiology at the community level has an important guiding role in zoonotic disease control programmes where genetic markers are suitably variable to unravel the dynamics of local transmission. We evaluated the molecular diversity of *Trypanosoma cruzi*, the etiological agent of Chagas disease, in southern Ecuador (Loja Province). This kinetoplastid parasite has traditionally been a paradigm for clonal population structure in pathogenic organisms. However, the presence of naturally occurring hybrids, mitochondrial introgression, and evidence of genetic exchange in the laboratory question this dogma.

**Methodology/Principal Findings:**

Eighty-one parasite isolates from domiciliary, peridomiciliary, and sylvatic triatomines and mammals were genotyped across 10 variable microsatellite loci. Two discrete parasite populations were defined: one predominantly composed of isolates from domestic and peridomestic foci, and another predominantly composed of isolates from sylvatic foci. Spatial genetic variation was absent from the former, suggesting rapid parasite dispersal across our study area. Furthermore, linkage equilibrium between loci, Hardy-Weinberg allele frequencies at individual loci, and a lack of repeated genotypes are indicative of frequent genetic exchange among individuals in the domestic/peridomestic population.

**Conclusions/Significance:**

These data represent novel population-level evidence of an extant capacity for sex among natural cycles of *T. cruzi* transmission. As such they have dramatic implications for our understanding of the fundamental genetics of this parasite. Our data also elucidate local disease transmission, whereby passive anthropogenic domestic mammal and triatomine dispersal across our study area is likely to account for the rapid domestic/peridomestic spread of the parasite. Finally we discuss how this, and the observed subdivision between sympatric sylvatic and domestic/peridomestic foci, can inform efforts at Chagas disease control in Ecuador.

## Author Summary

*Trypanosoma cruzi* is transmitted by blood sucking insects known as triatomines. This protozoan parasite commonly infects wild and domestic mammals in South and Central America. However, triatomines also transmit the parasite to people, and human infection with *T. cruzi* is known as Chagas disease, a major public health concern in Latin America. Understanding the complex dynamics of parasite spread between wild and domestic environments is essential to design effective control measures to prevent the spread of Chagas disease. Here we describe *T. cruzi* genetic diversity and population dynamics in southern Ecuador. Our findings indicate that the parasite circulates in two largely independent cycles: one corresponding to the sylvatic environment and one related to the domestic/peridomestic environment. Furthermore, our data indicate that human activity might promote parasite dispersal among communties. This information is the key for the design of control programmes in Southern Ecuador. Finally, we have encountered evidence of a sexual reproductive mode in the domestic *T. cruzi* population, which constitutes a new and intriguing finding with regards to the biology of this parasite.

## Introduction

Chagas disease, caused by the protozoan *Trypanosoma cruzi*, is the most important parasitic infection in Latin America [1]. An estimated 10 million people carry the infection, while another 90 million live at risk [2]. This vector-borne zoonosis causes severely debilitating and potentially deadly disease in more than a third of infected people [3]. Mucosal or abrasion contact with the infected faeces of hematophagous triatomine bugs constitutes the major mode of transmission [2].

Chagas disease is endemic to several regions in Ecuador, including the warm inter-Andean valleys of the southern province of Loja, where the main vectors are *Rhodnius ecuadoriensis*, *Triatoma carrioni*, *Panstrongylus chinai*, and *Panstrongylus rufotuberculatus* [4], [5]. Loja Province is currently targeted by the Ecuadorian Chagas Disease Control Program. Complementing disease prevention efforts, recent progress has been made in understanding local vector dynamics [5]–[7]. However, parasite molecular epidemiology could also play a role in guiding effective intervention measures.

Molecular diversity was first recognised in *T. cruzi* in the early 1970s [8]. Six major genetic subdivisions, known as discrete typing units (DTUs), are currently recognized (TcI–TcVI [9]), with distributions loosely defined by geography, transmission cycle, and ecology [1]. TcI predominates in northern South America, causes significant human disease [10], [11] and occurs in both domestic and sylvatic cycles of parasite transmission. Of major interest to those planning sustainable control strategies in this region is the extent to which these cycles are connected [12]–[14]. The provision of such data relies on the evaluation of molecular diversity ‘hidden’ at the sub-DTU level [15]–[17].

Hypervariable molecular markers, like microsatellites, have given new and unprecedented insight into the population genetics of other important parasitic zoonoses [18]–[22]. For the first time, specific hypotheses regarding parasite dispersal and reproduction can be addressed. However, the validity of molecular epidemiological data depends heavily on study design. Numerous confounders, including biased sampling (e.g., sampling only one host in a heteroxenous transmission system [23]), population subdivision in both space and time (leading to Wahlund effects [24]), and low sample size all influence the estimation of key population genetic parameters. Historically, such biases have acted as an impediment to obtaining useful epidemiological information from parasite molecular data, and, particularly in *T. cruzi*, to resolving the frequency of sex in natural populations.

Here we present microsatellite data for 10 variable loci amplified from a large number of TcI isolates collected from domestic, peridomestic, and sylvatic hosts and vectors in and around several adjacent communities in Loja Province, Ecuador. We evaluate evidence for genetic subdivision between transmission cycles, anthropogenic dispersal of parasites between communities, and panmixia among a subset of strains.

## Methods

### Study area and sampling

**Figure 1:**
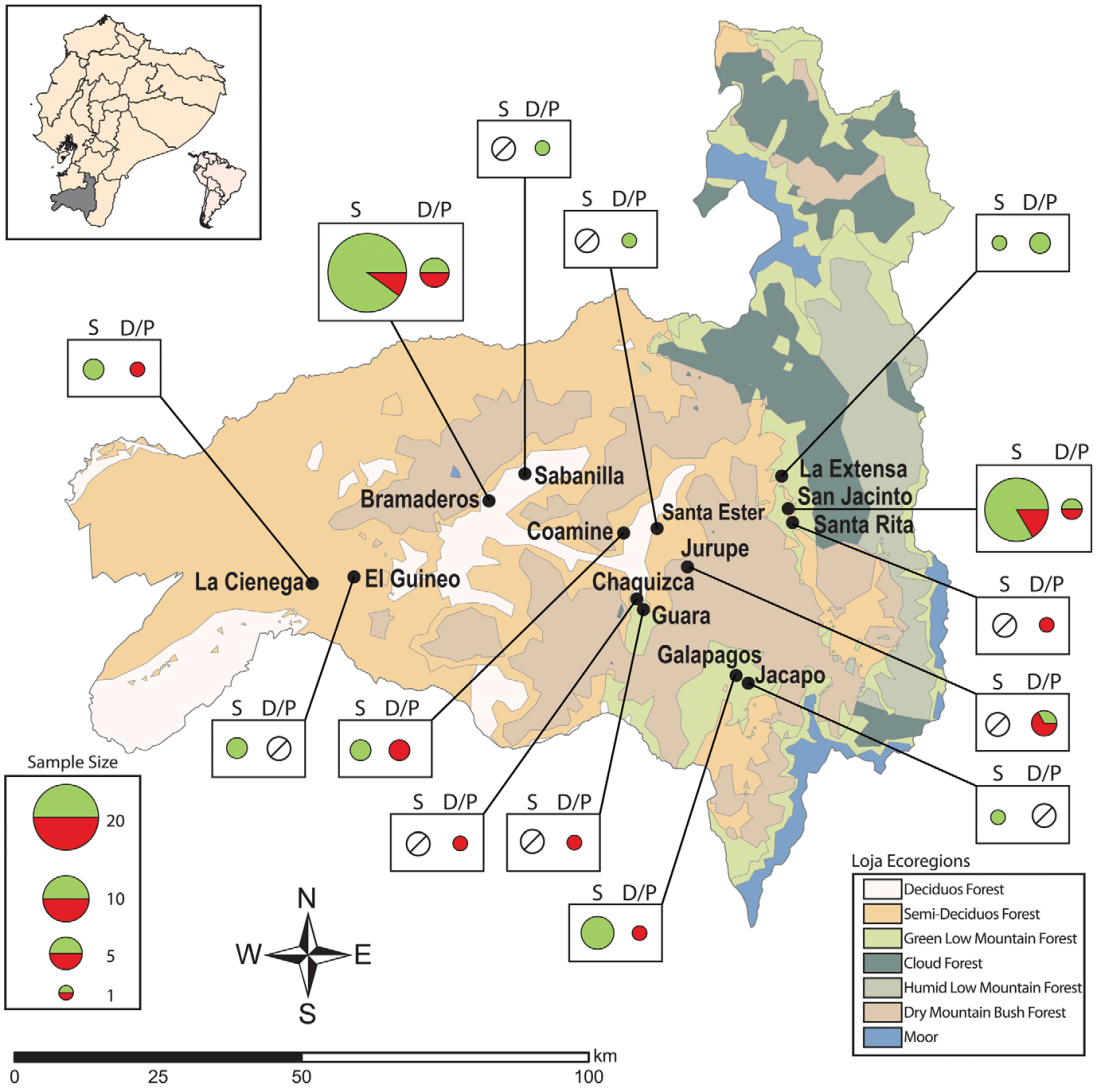
Distribution of genotypes in communities and transmission cycles in Loja Province. Black dots indicate locations from which isolates were obtained. Colored circles indicate proportion of *LOJA*_Sylv_ (green) and *LOJA*_Dom/Peri_ (red) genotypes (see Figure 2) among *T. cruzi* isolates from foci designated as sylvatic (S) or domestic/peridomestic (D/P). Crossed circles indicate absence of isolates from a particular location.The area of each pie chart represents the number of samples. *LOJA*_Dom/Peri_ is more common among D/P foci, largely irrespective of community. However, some cross-propagation between transmission cycles is evident. Insert shows in gray the location of Loja province within Ecuador and of Ecuador within South America.

Sixteen communities in Loja Province, southern Ecuador, were sampled (Figure 1). These communities were located at altitudes less than 2,200 m and were representative of the ecological diversity of the province. Trypanosomes were isolated from triatomines and small mammals (rodents and opossums) captured at domestic (within dwellings), peridomestic (near dwellings and/or associated with human activities, e.g., crop stores, chicken roosts, wood and rock piles), and sylvatic (more than 20 meters from dwellings) foci (Table S1). Written informed consents from the head of the houses for domiciliary bug searches and capture of mammals near houses were obtained. These documents have been approved by the institutional review board from National Institute of Health (NIH), Ohio University (OU) and Pontifical Catholic University of Ecuador (PUCE). Vertebrates were euthanized to obtain samples; all procedures were carried out in strict accordance with the protocol approved by the Ohio University Institutional Animal Care and Use Committee (IACUC). The Ohio University IACUC adheres to the guidelines in the United States Government Code of Federal Regulations (CFR), Title 9, Chapter 1, Subchapter A- Animal Welfare Parts 1–3 and the United States Health Research Extension Act of 1985, Public Law 99–158 “Animals in Research”.

### Molecular identification of *T. cruzi* and lineage genotyping

Trypanosome species was determined by PCR amplification of the kinetoplast minicircle region as in Vallejo et al. [25]. Discrete Typing Units (DTU) genotyping was achieved by assaying a combination of three nuclear loci as described by Lewis et al. [26].

### Microsatellite analysis

**Figure 2:**
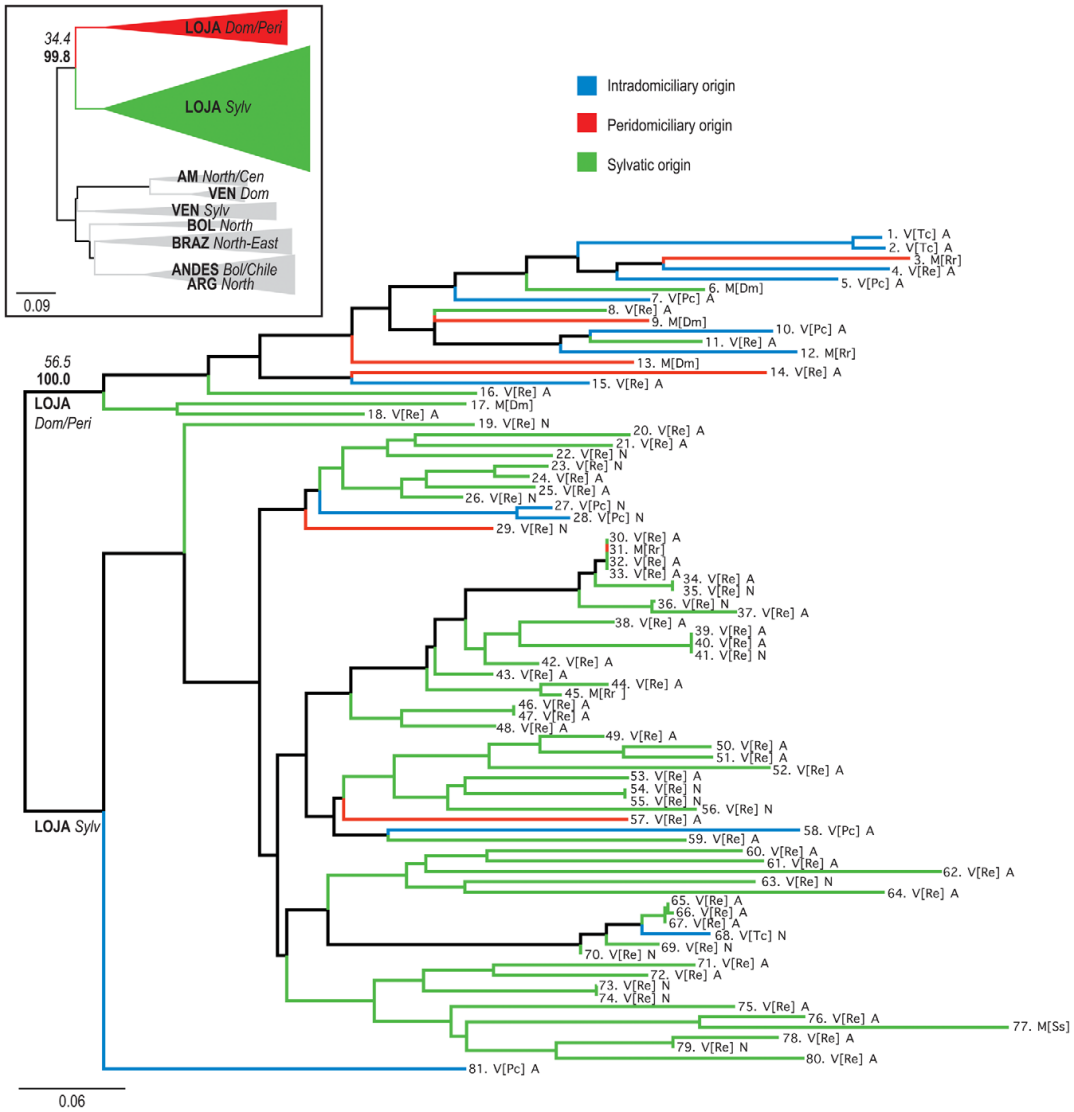
Neighbor-joining tree based on *D*_AS_ values from 10 microsatellite loci. Continental-scale affiliations of Loja TcI strains are shown in the insert figure and the local subdivision between transmission cycles is shown in the main figure. *T. cruzi D*_AS_ values correspond to the mean of 1,000 random diploid re-samplings of the dataset. Solid grey triangles indicate samples from other regions of South America while colored triangles indicate Ecuadorian populations (*LOJA*_Dom/Peri_ and *LOJA*_Sylv_). Branch color codes indicate capture environment. Blue: domicile; Red: peridomicile; Green: sylvatic. Sample codes were defined as follows: M: mammal; Dm: *Didelphis marsupialis*; Rr: *Rattus rattus*; Ss: *Sciureus stramineus*; V: vector; Pc: *Panstrongylus chinai*; Tc: *Triatoma carrioni*; Re: *Rhodnius ecuadoriensis*; A: adult; N: nymph. Values in italics correspond to bootstraps (%) over 10,000 trees drawn from 100 randomly sampled diploid datasets. Values in bold correspond to topological stability (percentage congruent trees) over 1,000 randomly sampled diploid datasets (see *Methods*).

Ten previously identified polymorphic microsatellite loci were studied (Table S2) [16]. These loci are distributed across seven *T. cruzi* chromosomes and include two groups of physically linked markers [27]. Allelic products were amplified using previously described reaction conditions [16]. Allele sizes were determined using an automated capillary sequencer (AB3730, Applied Biosystems, UK) in conjunction with a fluorescently tagged size standard and were manually checked for errors. All isolates were typed “blind” to control for user bias. By reference to a representative panel of strains, no cross reactivity was identified between *T. rangeli* and the microsatellite primers used in this study. Population-level genetic diversity was assessed using sample size corrected allelic richness (A_r_) in FSTAT 2.9.3.2 [28] and number of private (population specific) alleles per locus (PA). *F*_IS_, a measure of the distribution of heterozygosity within and between individuals, was estimated per locus per population in FSTAT 2.9.3.2. *F*_IS_ can vary between −1 (all loci heterozygous for the same alleles) and +1 (all loci homozygous for different alleles). *F*_IS_ = 0 indicates Hardy-Weinberg allele proportions. The extent of population subdivision between isolates from different transmission cycles was estimated using (*F*_ST_) in ARLEQUIN v3.1 and statistical significance assessed via 10,000 random permutations of alleles between populations [29]. Similarly, within-population subdivision was examined in ARLEQUIN v3.1, in this case using a hierarchal Analysis of Molecular Variance (AMOVA). Population-level heterozygosity indices were also calculated in ARLEQUIN v3.1 and associated significance levels for p values derived after sequential Bonferroni correction to minimise the likelihood of Type 1 errors [30]. Individual-level pair-wise distances were estimated using *D*_AS_ (1-proportion of shared alleles at all loci / *n*) [31] under an IAM and δμ^2^ [32] under an SMM in MICROSAT [33]. *D*_AS_ values form the basis of the dendrogram in Figure 2. To accommodate multi-allelic loci, a script was written in Microsoft Visual Basic to make multiple random diploid re-samplings of each multilocus profile (software available on request). Individual-level genetic distances were calculated as the mean across multiple re-sampled datasets. A Mantel's test for the effect of isolation by distance within populations (pair-wise genetic *vs.* geographic distance) was implemented in Genelax 6 using 10,000 random permutations [34]. Linkage disequilibrium indices, pair-wise (R_GGD_) and multilocus (I_A_), were calculated in LINKDOS [35] and MULTILOCUS1.3b [36], respectively. Multiple diploid re-samplings were also made to evaluate the influence of multi-allelic loci on I_A_, the results of which are shown in Table 1. Assignment of individuals to populations was made by reference to the topology of the *D*_AS_ derived tree. Secondarily, this model-free population assignment was corroborated using STRUCTURE (Figure S1) [37]. Sample affiliations are listed in Table S1.

**Table 1:** Population genetic parameters for domestic/peridomestic and sylvatic populations of *T cruzi* in Loja Province, Ecuador.

Population	G/N	PL	PA	MA/S	A_r_±SE	H_O_ *	H_E_ *	% PL H_E_ **	% PL H_D_ †	F_IS_±SE††	I_A_ ‡	Median I_A_ *P*-value‡	% PL PLD‡‡
**Domestic/Peridomestic**	18/18	9	0.8	0.67	3.851±0.527	0.478	0.478	0	0	0.003±0.084	0.170	0.13	5.5
**Sylvatic**	55/63	9	2.0	0.24	4.583±0.504	0.367	0.457	11.1	44.4	0.184±0.098	0.562	<0.001	38.5

N = Number of isolates in population.

G = Number of multilocus genotypes per population.

PL = Number of polymorphic loci.

PA = Mean number of private alleles per locus.

MA/S = Mean number of multiple (3+) alleles per sample.

A_r_ = Allelic richness as a mean over loci ± standard error, calculated in FSTAT (28).

*Mean observed and expected heterozygosity across all loci, calculated in ARLEQUIN v3.1 (29).

**Proportion of loci showing a significant excess in heterozygosity after a sequential Bonferroni correction. Calculated in ARLEQUIN v3.1 (29).

**†:** Proportion of loci showing significant deficit in heterozygosity after a sequential Bonferroni correction. Calculated in ARLEQUIN v3.1(29).

**††:** Mean FIS over loci ± standard error, calculated in FSTAT (28).

**‡:** Calculated in Multilocus v1.3; p-value derived through comparison to a null distribution of 1,000 randomizations. Median values taken from 1,000 diploid resamplings of the multiallelic dataset.

**‡‡:** Proportion of loci demonstrating significant pair-wise linkage (coefficient of correlation *R*_GGD_, after a sequential Bonferroni correction. Calculated in LINKDOS(35).

## Results

Eighty-one isolates of *T. cruzi* were obtained from triatomines and mammals. All were genotyped as TcI. Kinetoplast analysis detected the presence of mixed infection with *T. rangeli* in nine isolates from the sylvatic environment (Table S1). In total, a dataset of 1,637 alleles was derived across all loci, excluding missing data (Table S3). Multiple (≥3) alleles were observed at 3.08% of loci.

### Isolate clustering and population subdivision by transmission cycle

We evaluated patterns of clustering and subdivision among parasite strains in the Loja samples based upon their microsatellite profiles. To identify genetically distinct groups we relied on three lines of evidence: neighbor-joining analysis based on pair-wise genetic distance; model-based population assignment (STRUCTURE); and the statistical significance of the fixation index *F*_ST_.The deepest and most robust (56.5%) internal branching within the neighbor-joining tree constructed from pair-wise genetic distance values (*D*_AS_) supported the delineation of two populations (Figure 2 and Table 1). No pattern or diversification by host or vector was observed within these populations. The observed bipartite subdivision was unaffected by the presence of multi-allelic loci (100% congruence, Figure 2) and was used as a means to define the populations examined in later analyses (See Table 1). Sample allocation between these two populations was exactly corroborated by the optimal number of clusters (*k*) derived using STRUCTURE software as defined by Evanno et al. [37] by Δ*k* (Figure S1). One population, henceforth called *LOJA*_Dom/Peri_, was predominantly composed of isolates from domestic and peridomestic foci, the other, henceforth *LOJA*_Sylv_, of isolates from the sylvatic environment. Estimates of genetic subdivision (*F*_ST_) between *a priori* populations (transmission cycle defined) corroborated this pattern of dispersal. No evidence for subdivision existed between domestic and peridomestic isolates (*F*_ST_ = 0.027, p = 0.354), whereas subdivision between these populations (grouped) and sylvatic samples was pronounced (*F*_ST_ = 0.212, p<0.0001). Naturally, reassignment of outliers to their “correct” genetic groups according to neighbor-joining and STRUCTURE analyses further inflated the latter estimate (*F*_ST_ *LOJA*_Dom/Peri_−*LOJA*_Sylv_ = 0.397, p<0.0001). These outliers are evidence for some, albeit limited, parasite dispersal between domestic/peridomestic transmission cycles and sylvatic transmission cycles as evident in Figure 1 and 2.

### Genetic diversity

Following the identification of two genetically distinct groups of parasite strains circulating in this endemic area, the genetic diversity of each was evaluated and compared. Estimates of allelic richness (A_r_) did not demonstrate dramatic difference between *LOJA*_Dom/Peri_ and *LOJA*_Sylv_ (Table 1); both populations showed considerable genetic diversity. More private alleles per locus (PA) were found in the larger and marginally more diverse sylvatic population (PA = 2.0; Table 1). In conjunction with its apparent genetic distance from other South American TcI populations (Figure 2), the lack of private alleles within *LOJA*_Dom/Peri_ (PA = 0.8) suggests diversification of this population from a local source.

### Geographic dispersal within populations

**Figure 3:**
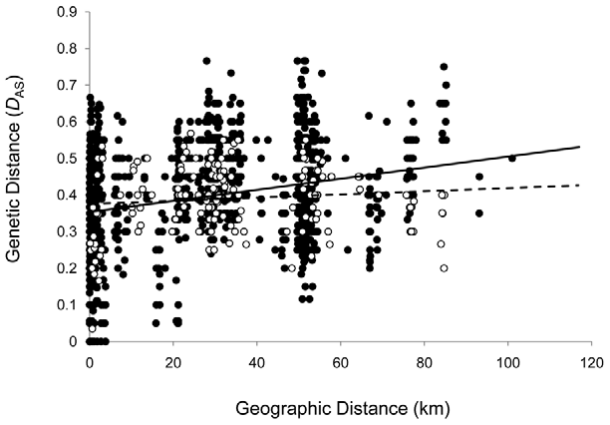
Spatial genetic analysis among *T. cruzi* isolates from *LOJA*_Sylv_ and *LOJA*_Dom/Peri_. A spatial structure was evident within *LOJA*_Sylv_ isolates, while no spatial genetic structure was found among *LOJA*_Dom/Peri_ isolates. Genetic (*D*_AS_) and geographic (km) distance were compared. Closed circles and line correspond to samples from *LOJA*_Sylv_ (R_XY_ = 0.265, p = 0.000; Slope = 0.0015±0.0001 (SE)); open circles and dashed line correspond to samples from *LOJA*_Dom/Peri_ (R_XY_ = 0.100, p = 0.164; Slope = 0.0004±0.0004 (SE)). Equivalent statistics under δμ^2^ are *LOJA*_Sylv_ R_XY_ = 0.177, p = 0.001; *LOJA*_Dom/Peri_ R_XY_ = −0.052, p = 0.384.

In light of the role played by transmission cycles in structuring the local parasite population, we compared the rate of parasite dispersal within *LOJA*_Dom/Peri_ with that within *LOJA*_Silv_. This rate is inversely proportional to the amount of spatial structure (or isolation by distance (IBD)) in the population. Interestingly, tests for IBD among individuals from *LOJA*_Dom/Peri_ and *LOJA*_Sylv_ showed statistically significant and epidemiologically important differences between these two populations. Infinite allele models (IAMs) of microsatellite mutation intrinsically overestimate genetic distances between closely related isolates as compared to stepwise mutational models (SMMs). To circumvent possible bias we chose to test for IBD under both. Strong evidence for spatial structure in *LOJA*_Sylv_ existed regardless (*D*_AS_−R_XY_ = 0.265, *P*<0.0001; δμ^2^−R_XY_ = 0.177, p = 0.001). Among isolates from *LOJA*_Dom/Peri_, no spatial structure was evident from either measure (*D*_AS_−R_XY_ = 0.100, p = 0.164; δμ^2^−R_XY_ = −0.05, p = 0.384). Results are summarised in Figure 3 and strongly suggest more rapid parasite dispersal among domestic and peridomestic foci than that occurring between sylvatic locales at the same spatial scale.

### Compelling evidence for sex in *LOJA*_Dom/Peri_

Several approaches were employed to estimate the rate of genetic recombination within the parasite populations identified in Loja. Multiple indicators suggested frequent sex among trypanosomes of *LOJA*_Dom/Peri_. Pair-wise inter-locus linkage (*R*_GGD_) was infrequent (5.5%; Table 1) even among physically linked loci (3/4 physically linked locus pairs, those on the same chromosome, were not statistically linked) and despite abundant allelic diversity available within this population for inter-correlation (the statistical power of *R*_GGD_ drops dramatically with decreasing population-level genetic diversity). Infrequent pair-wise linkage is consistent with the lack of significance attributable to the index of association (I_A_) (median p = 0.13, *P*≥0.05 in 89% of 1000 resampled diploid datasets; Table 1), and with the null hypothesis of random mating that must be assumed. Additionally, tests for deficit or excess of heterozygosity in this population showed no significant deviation from Hardy-Weinberg expectations, reflected by mean values for the inbreeding coefficient (*F*_IS_) across loci that approximate zero (Table 1). Finally, repeated multilocus genotypes, indicative of clonal reproduction, were absent from this population while present in *LOJA*_Sylv._ Other aspects of *LOJA*_Sylv_ diversity pointed to predominant clonality, especially strong pair-wise (38.5% of locus pairs) and multilocus linkage (I_A_ *P*<0.001) in all diploid resampled datasets (Table 1), but also strong deviation from Hardy-Weinberg levels of heterozygosity under all three measures employed (Table 1). Consistent with spatial structure identified in this population, however, an AMOVA conducted across isolates from San Jacinto and Bramaderos, which make up the majority of *LOJA*_Sylv_ strains (Figure 1 and Table S1), did demonstrate significant but weak *F*_ST_ (*F*_ST_ = 0.173, *P*<0.0001, 16,000 permutations), evidence that a Wahlund effect could be depressing heterozygosity. Correspondingly, estimates of linkage disequilibrium might also be somewhat inflated by subdivision in this population [38], and it is difficult to reject the possibility that recombination may occur in the sylvatic populations at a micro-geographic scale.

## Discussion

This study constitutes a first attempt to understand the population dynamics of *T. cruzi* at a local scale using high-resolution molecular markers. The sample includes isolates from different transmission cycles, vectors, hosts, and adjacent communities. This arrangement aims to minimise sample bias and maximise the resulting molecular epidemiological inference. However, all field studies are affected by the natural abundance of hosts and vectors in different transmission cycles, and we cannot claim a perfect dataset. Nonetheless, we can report strong evidence for parasite diversification by transmission cycle, human involvement in parasite dispersal, and the possibility of sex in one parasite population.

The presence of the *T cruzi* lineage I in southern Ecuador is consistent with reports of this DTU throughout northern South America [10], [39], [40]. In our study, as in other studies, sub-DTU level diversity of the parasite occurred independently of vector and host [16], [17], [41]. Instead, we found evidence that transmission cycle (domestic, peridomestic, or sylvatic) is likely to be the major driver behind parasite differentiation, apparently a phenomenon common to *T. cruzi* populations across much of northern South America [15], [16] but never before studied on a local scale. On the basis of our data, we suggest that widespread, internationally distributed TcI subgroups associated with specific transmission cycles may not exist. A lack of connectivity between *LOJA*_Dom/Peri_ and domestic TcI from Venezuela, *VEN*_Dom_, (Figure 2) exemplifies this. Furthermore, clear cross-propagation of parasites between transmission cycles (Figure 2) and few private alleles in *LOJA*_Dom/Peri_ (Table 1) suggests that these domestic groups are likely to emerge and diversify from local sylvatic sources.

*T. cruzi* is the only stercorarian trypanosome of medical importance [42]. Natural transmission efficiency by this route (contamination with vector feces) is very low. The rate of transmission from infected *Triatoma infestans* to humans in Argentina, for example, is estimated at approximately one in 650 bites [43]. As with *R. prolixus* in Venezuela [14], *R. ecuadoriensis*, a major disease vector in Loja, occurs at high frequency in both domestic and sylvatic locales [7]. Our data suggest that even if vector invasion from sylvatic foci is common, as in Venezuela [14], associated transmission of parasites to domestic foci is too infrequent to break up population subdivision. Where cross-propagation does occur, circumstantial evidence incriminates synanthropic mammals as the link between transmission cycles. *Didelphis marsupialis* infected with parasites from the *LOJA*_Dom/Peri_ group were found at both peridomestic (Isolate Numbers (IN) 9 and 13, Figure 2) and sylvatic locales (IN 6 and 17, Figure 2). Furthermore, a *R. rattus* individual captured at a peridomestic site was found infected with a *LOJA*_Sylv_ strain (IN 31, Figure 2). Finally *P. chinai* and *T. carrioni* adults and nymphs, so far thought to be exclusively domestic and peridomestic triatomine species in Loja (IN 27,28,58,68 and 81, Figure 2) [5], were found infected with a *LOJA*_Sylv_ strain, likely as a result of contact with invasive sylvatic mammals. This blurring of the lines between transmission cycles is likely to mirror local environmental change, where human activity is driving land-use transformation.

Parasite sampling in Loja was undertaken across an area only 50 km in radius (Figure 1). However, this area encompassed several ecological zones punctuated by high mountains (>2,500 m in elevation) and deep interconnecting valleys. Spatial genetic diversification among sylvatic isolates is an expected outcome given such barriers to host and vector migration (Figure 1 and 3). Conversely, parasites belonging to the *LOJA*_Dom/Peri_ group lack this signature, a finding possibly linked to rapid anthropogenic dispersal in the form of infected individuals, livestock, or passively transported vectors and/or small peridomestic mammals.

*T. cruzi* has, until recently, been a paradigm for clonal population structure in pathogenic organisms [44], [45]. However, the presence of naturally occurring hybrids [46], mitochondrial introgression [46], and a capacity for genetic exchange in the laboratory [47] has challenged this dogma. The frequent observation of linkage disequilibrium in *T. cruzi* may partially stem from cryptic population subdivision (temporal, spatial, and/or genetic) to which linkage statistics are intrinsically sensitive [38]. Frustratingly, if assignment software with intrinsic Hardy-Weinberg assumptions (e.g., STRUCTURE [48] or BAPS [49]) is used to account for subdivision prior to linkage analysis, the resulting populations will be sorted to maximise adherence to Hardy-Weinberg allelic frequencies, with artifactual sexuality a possible result [21], [50]. Fortunately, in our study, the status of *LOJA*_Dom/Peri_ as a stable deme is corroborated by distance-based, model-free assignment, as well as STRUCTURE. In conjunction with Hardy-Weinberg allele frequencies at individual loci, we consider, therefore, that linkage equilibrium among isolates from *LOJA*_Dom/Peri_ represents strong evidence for frequent genetic exchange among field isolates of *T. cruzi*. We believe that the relatively small sample size of *LOJA*_Dom/Peri_ (n = 18) does not affect this conclusion, partly due to the ample genetic diversity present in this popualtion, but also because the lack of spatial subdivision in this group suggests frequent contact and opportunity for mixis. Thus it constitutes exactly the group of strains between which genetic exchange might be expected to occur. We cannot rule out the possibility that genetic exchange may also occur in the sylvatic cycle, if the role that substructure found in *LOJA*_Sylv_ played in inflating linkage statistics I_A_ and R_GGD_ could be taken into account. However, more focused high-density sample collection from multiple individual localities would be required to address such a hypothesis. Furthermore, we cannot infer the cellular mechanism of genetic recombination events on the basis of these data. Hardy-Weinberg allelic allelic frequencies are consistent with classical meiosis. However, the lack of haploid life stages so far observed in *T. cruzi* are not consistent with classical meiosis, nor are the genetic exchange events so far observed *in vitro* [47].

Molecular epidemiology at this scale has an important guiding role to play in Chagas disease control programmes. Future efforts in Loja province must account for inter-domiciliary and inter-community parasite dispersal. This includes sustained surveillance and coordinated region-wide spraying campaigns to eliminate local vector re-invasion sources and community education to target passive triatomine dispersal routes. It is also clear that the role of synanthropic mammals cannot be overlooked as these represent an important potential link between sylvatic and domestic foci.

We have shown that microsatellite markers, adequate sample sizes, and associated population statistics give fundamental insight into the genetic exchange in *T. cruzi*. Our results, skewed toward samples from the vector, intuitively imply that the vector may be a site of genetic exchange, as is the case for *T. brucei* [51] and *Leishmania major* [52]. The data also indicate, not surprisingly, that the majority of events probably occur within a *T. cruzi* lineage between epidemiologically linked strains, and these events have therefore historically been difficult to detect. The intriguing mechanisms of genetic exchange in *T. cruz*i warrant further investigation of their functional, adaptive, and epidemiological significance.

## Supporting Information

Figure S1 Clustering of populations based on STRUCTURE analysis. Results from structure analysis corresponded with the structure defined by *D*_AS_ values. Both analyses identified two distinct populations among Ecuadorian samples. Ten replicates per value *k* were made assuming a no-admixture model, and with a burn in of 100,000 followed by 1,000,000 interactions of the algorithm. Delta *k* calculated according to Evanno et al. [37].
(6.59 MB TIF)

Table S1 Location, habitat, host, and lineage of *Trypanosoma* isolated in Loja Province, Ecuador.
(0.15 MB DOC)

Table S2 *Trypanosoma cruzi* primers employed in this study.
(0.04 MB DOC)

Table S3 Allele sizes (in base pairs) from 10 loci analyzed (fluorescent dye) from 81 *T. cruzi* isolates from Loja Province, Ecuador.
(0.30 MB DOC)
