# Supplementary figures and images for: Sex, Subdivision, and Domestic Dispersal of Trypanosoma cruzi Lineage I in Southern Ecuador
Source: PLoS Negl Trop Dis. 2010 Dec 14;4(12):e915. doi: 10.1371/journal.pntd.0000915 (PMC3001902; doi:10.1371/journal.pntd.0000915)

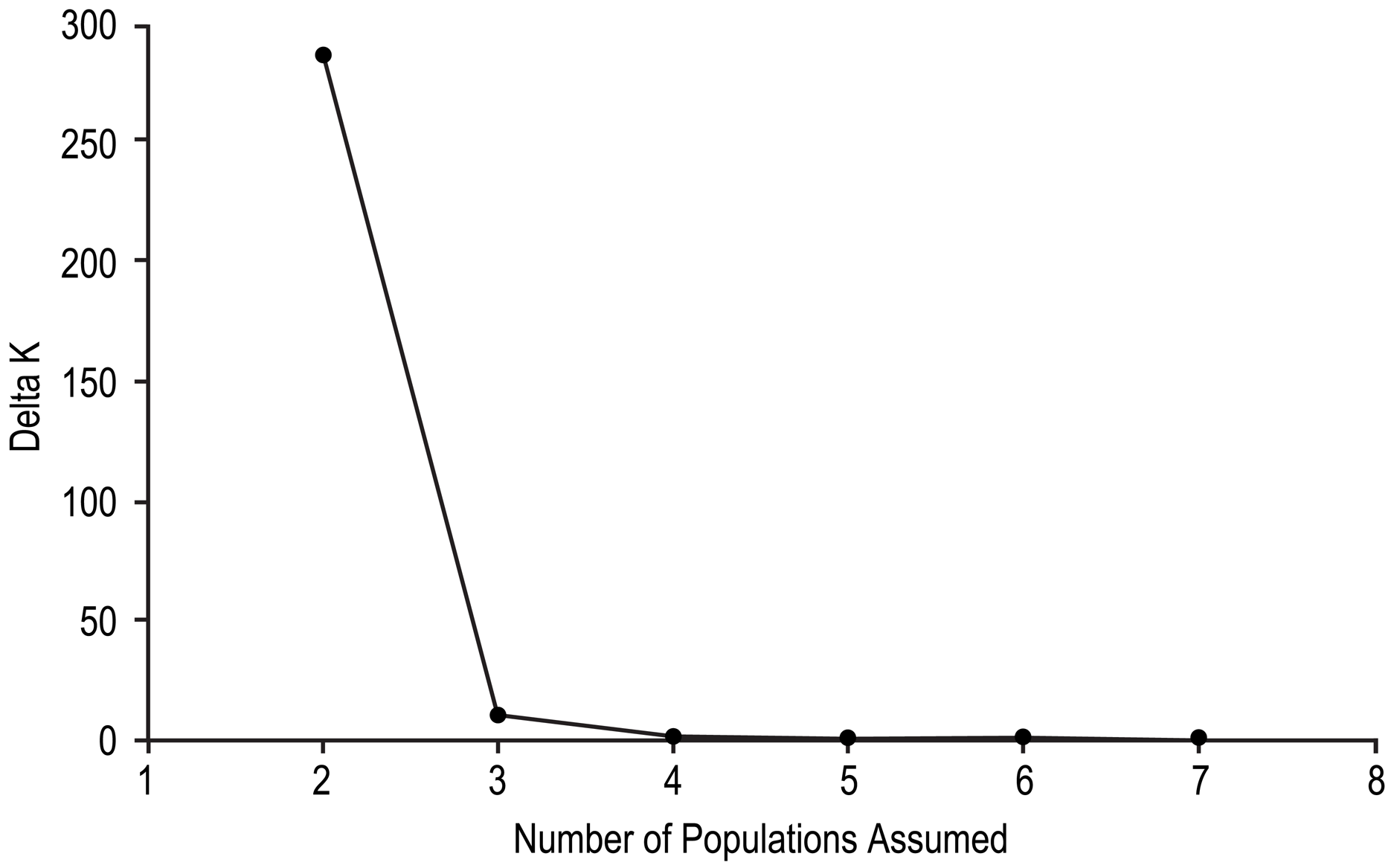

Supplement: Figure S1 — Clustering of populations based on STRUCTURE analysis. Results from structure analysis corresponded with the structure defined by D AS values. Both analyses identified two distinct populations among Ecuadorian samples. Ten replicates per value k were made assuming a no-admixture model, and with a burn in of 100,000 followed by 1,000,000 interactions of the algorithm. Delta k calculated according to Evanno et al. [37]. (6.59 MB TIF) [file pntd.0000915.s001.tif]
