# Supplementary material for: Sex, Subdivision, and Domestic Dispersal of Trypanosoma cruzi Lineage I in Southern Ecuador
Source: PLoS Negl Trop Dis. 2010 Dec 14;4(12):e915. doi: 10.1371/journal.pntd.0000915 (PMC3001902; doi:10.1371/journal.pntd.0000915)
Supplement: Table S1 — Location, habitat, host, and lineage of Trypanosoma isolated in Loja Province, Ecuador. (0.15 MB DOC) [file pntd.0000915.s002.doc]

**Table S1.** Location, habitat, host, and lineage of *Trypanosoma* isolated in Loja Province, Ecuador.

|  | **Sample Code** | **Structure/ *D*_AS_ population** | **Habitat of capture** | **Mammalian host/vector origin** | **Community** | **County** | **Latitude^§^** | **Longitude^§^** | ***T. cruzi* lineage (kinetoplast trypanosome identification)** |
| --- | --- | --- | --- | --- | --- | --- | --- | --- | --- |
| 1 | TJU034 | *LOJA*_Dom/Peri_ | dom | *Triatoma carrioni* | Jurupe | Gonzanamá | -4,183 | -79,521 | TcI (*T. cruzi*) |
| 2 | TJU030 | *LOJA*_Dom/Peri_ | dom | *Triatoma carrioni* | Jurupe | Gonzanamá | -4,188 | -79,517 | TcI (*T. cruzi*) |
| 3 | BR6383 | *LOJA*_Dom/Peri_ | peri | *Rattus rattus (black rat)* | Bramaderos | Paltas | -4,072 | -79,834 | TcI (*T. cruzi*) |
| 4 | TBR123 | *LOJA_Dom/Peri_* | dom | *Rhodnius ecuadoriensis* | Bramaderos | Paltas | -4,079 | -79,825 | TcI (*T. cruzi*) |
| 5 | GA817 | *LOJA*_Dom/Peri_ | dom | *Panstrongylus chinai* | Guara | Calvas | -4,254 | -79,58 | TcI (*T. cruzi*) |
| 6 | BR1331 | *LOJA*_Dom/Peri_ | sylv | *Didelphis marsupialis (opossum)* | Bramaderos | Paltas | -4,078 | -79,807 | TcI (*T. cruzi*) |
| 7 | CE682 | *LOJA*_Dom/Peri_ | dom | *Panstrongylus chinai* | Coamine | Paltas | -4,123 | -79,605 | TcI (T. cruzi) |
| 8 | SJ778 | *LOJA*_Dom/Peri_ | sylv | *Rhodnius ecuadoriensis* | San Jacinto | Gonzanamá | -4,106 | -79,347 | TcI (T. cruzi) |
| 9 | SJ1147 | *LOJA*_Dom/Peri_ | peri | *Didelphis marsupialis (opossum)* | San Jacinto | Gonzanamá | -4,094 | -79,349 | TcI (*T. cruzi*) |
| 10 | RT672 | *LOJA*_Dom/Peri_ | dom | *Panstrongylus chinai* | Santa Rita | Gonzanamá | -4,116 | -79,348 | TcI (*T. cruzi*) |
| 11 | SJ713 | *LOJA*_Dom/Peri_ | sylv | *Rhodnius ecuadoriensis* | San Jacinto | Gonzanamá | -4,096 | -79,344 | TcI (*T. cruzi* / *T. rangeli*) |
| 12 | CG1232 | *LOJA*_Dom/Peri_ | dom | *Rattus rattus (black rat)* | La Ciénega | Celica | -4,21 | -80,099 | TcI (*T. cruzi*) |
| 13 | CE1126 | *LOJA*_Dom/Peri_ | peri | *Didelphis marsupialis (opossum)* | Coamine | Paltas | -4,135 | -79,614 | TcI (*T. cruzi*) |
| 14 | TGL004 | *LOJA*_Dom/Peri_ | peri | *Rhodnius ecuadoriensis* | Galápagos | Quilanga | -4,353 | -79,432 | TcI (*T. cruzi*) |
| 15 | CQ800 | *LOJA*_Dom/Peri_ | dom | *Rhodnius ecuadoriensis* | Chaquizhca | Calvas | -4,237 | -79,599 | TcI (*T. cruzi*) |
| 16 | SJ1083 | *LOJA*_Dom/Peri_ | sylv | *Rhodnius ecuadoriensis* | San Jacinto | Gonzanamá | -4,098 | -79,343 | TcI (*T. cruzi*) |
| 17 | BR1329 | *LOJA*_Dom/Peri_ | sylv | *Didelphis marsupialis (opossum)* | Bramaderos | Paltas | -4,078 | -79,807 | TcI (*T. cruzi*) |
| 18 | TBR199 | *LOJA*_Dom/Peri_ | sylv | *Rhodnius ecuadoriensis* | Bramaderos | Paltas | -4,077 | -79,808 | TcI (*T. cruzi*) |
| 19 | TGN081 | *LOJA*_Silv_ | sylv | *Rhodnius ecuadoriensis* | El Guineo | Celica | -4,196 | -80,034 | TcI (*T. cruzi*) |
| 20 | TBR193 | *LOJA*_Sylv_ | sylv | *Rhodnius ecuadoriensis* | Bramaderos | Paltas | -4,079 | -79,825 | TcI (*T. cruzi*) |
| 21 | TBR163 | *LOJA*_Sylv_ | sylv | *Rhodnius ecuadoriensis* | Bramaderos | Paltas | -4,075 | -79,809 | TcI (*T. cruzi*) |
| 22 | TBR230 | *LOJA*_Sylv_ | sylv | *Rhodnius ecuadoriensis* | Bramaderos | Paltas | -4,077 | -79,822 | TcI (*T. cruzi*) |
| 23 | TBR237 | *LOJA*_Sylv_ | sylv | *Rhodnius ecuadoriensis* | Bramaderos | Paltas | -4,077 | -79,809 | TcI (*T. cruzi*) |
| 24 | TBR153 | *LOJA*_Sylv_ | sylv | *Rhodnius ecuadoriensis* | Bramaderos | Paltas | -4,076 | -79,809 | TcI (*T. cruzi*) |
| 25 | TBR220 | *LOJA*_Sylv_ | sylv | *Rhodnius ecuadoriensis* | Bramaderos | Paltas | -4,078 | -79,807 | TcI (*T. cruzi*) |
| 26 | TBR218 | *LOJA*_Sylv_ | sylv | *Rhodnius ecuadoriensis* | Bramaderos | Paltas | -4,08 | -79,824 | TcI (*T. cruzi*) |
| 27 | EX320 | *LOJA*_Sylv_ | dom | *Panstrongylus chinai* | La Extensa | Catamayo | -4,045 | -79,361 | TcI (*T. cruzi*) |
| 28 | EX319 | *LOJA*_Sylv_ | dom | *Panstrongylus chinai* | La Extensa | Catamayo | -4,045 | -79,361 | TcI (*T. cruzi*) |
| 29 | TSA170 | *LOJA*_Sylv_ | peri | *Rhodnius ecuadoriensis* | Sabanilla | Paltas | -4,039 | -79,761 | TcI (*T. cruzi*) |
| 30 | SJ712 | *LOJA*_Sylv_ | sylv | *Rhodnius ecuadoriensis* | San Jacinto | Gonzanamá | -4,096 | -79,344 | TcI (*T. cruzi*) |
| 31 | SJ1141 | *LOJA*_Sylv_ | peri | *Rattus rattus (black rat)* | San Jacinto | Gonzanamá | -4,095 | -79,352 | TcI (*T. cruzi*) |
| 32 | SJ1079 | *LOJA*_Sylv_ | sylv | *Rhodnius ecuadoriensis* | San Jacinto | Gonzanamá | -4,106 | -79,347 | TcI (*T. cruzi*) |
| 33 | SJ1080 | *LOJA*_Sylv_ | sylv | *Rhodnius ecuadoriensis* | San Jacinto | Gonzanamá | -4,106 | -79,347 | TcI (*T. cruzi*) |
| 34 | SJ744 | *LOJA*_Sylv_ | sylv | *Rhodnius ecuadoriensis* | San Jacinto | Gonzanamá | -4,1 | -79,351 | TcI (*T. cruzi*) |
| 35 | SJ795 | *LOJA*_Sylv_ | sylv | *Rhodnius ecuadoriensis* | San Jacinto | Gonzanamá | -4,106 | -79,347 | TcI (*T. cruzi* / *T. rangeli*) |
| 36 | SJ1081 | *LOJA*_Sylv_ | sylv | *Rhodnius ecuadoriensis* | San Jacinto | Gonzanamá | -4,098 | -79,343 | TcI (*T. cruzi*) |
| 37 | SJ1127 | *LOJA*_Sylv_ | sylv | *Rhodnius ecuadoriensis* | San Jacinto | Gonzanamá | -4,105 | -79,347 | TcI (*T. cruzi*) |
| 38 | SJ737 | *LOJA*_Sylv_ | sylv | *Rhodnius ecuadoriensis* | San Jacinto | Gonzanamá | -4,1 | -79,351 | TcI (*T. cruzi*) |
| 39 | SJ718 | *LOJA*_Sylv_ | sylv | *Rhodnius ecuadoriensis* | San Jacinto | Gonzanamá | -4,096 | -79,344 | TcI (*T. cruzi* / *T. rangeli*) |
| 40 | SJ721 | *LOJA*_Sylv_ | sylv | *Rhodnius ecuadoriensis* | San Jacinto | Gonzanamá | -4,096 | -79,344 | TcI (*T. cruzi* / *T. rangeli*) |
| 41 | SJ717 | *LOJA*_Sylv_ | sylv | *Rhodnius ecuadoriensis* | San Jacinto | Gonzanamá | -4,096 | -79,344 | TcI (*T. cruzi* / *T. rangeli*) |
| 42 | SJ796 | *LOJA*_Sylv_ | sylv | *Rhodnius ecuadoriensis* | San Jacinto | Gonzanamá | -4,1 | -79,351 | TcI (*T. cruzi*) |
| 43 | SJ723 | *LOJA*_Sylv_ | sylv | *Rhodnius ecuadoriensis* | San Jacinto | Gonzanamá | -4,096 | -79,344 | TcI (*T. cruzi*) |
| 44 | SJ1082 | *LOJA*_Sylv_ | sylv | *Rhodnius ecuadoriensis* | San Jacinto | Gonzanamá | -4,098 | -79,343 | TcI (*T. cruzi*) |
| 45 | SJ1162 | *LOJA*_Sylv_ | sylv | *Rattus rattus (black rat)* | San Jacinto | Gonzanamá | -4,062 | -79,205 | TcI (*T. cruzi*) |
| 46 | TBR224 | *LOJA*_Sylv_ | sylv | *Rhodnius ecuadoriensis* | Bramaderos | Paltas | -4,08 | -79,824 | TcI (*T. cruzi*) |
| 47 | TBR238 | *LOJA*_Sylv_ | sylv | *Rhodnius ecuadoriensis* | Bramaderos | Paltas | -4,076 | -79,809 | TcI (*T. cruzi*) |
| 48 | SJ738 | *LOJA*_Sylv_ | sylv | *Rhodnius ecuadoriensis* | San Jacinto | Gonzanamá | -4,1 | -79,351 | TcI (*T. cruzi*) |
| 49 | TBR150 | *LOJA*_Sylv_ | sylv | *Rhodnius ecuadoriensis* | Bramaderos | Paltas | -4,076 | -79,809 | TcI (*T. cruzi*) |
| 50 | TBR155 | *LOJA*_Sylv_ | sylv | *Rhodnius ecuadoriensis* | Bramaderos | Paltas | -4,076 | -79,809 | TcI (*T. cruzi*) |
| 51 | TBR149 | *LOJA*_Sylv_ | sylv | *Rhodnius ecuadoriensis* | Bramaderos | Paltas | -4,076 | -79,809 | TcI (*T. cruzi*) |
| 52 | TBR215 | *LOJA*_Sylv_ | sylv | *Rhodnius ecuadoriensis* | Bramaderos | Paltas | -4,079 | -79,807 | TcI (*T. cruzi*) |
| 53 | TBR240 | *LOJA*_Sylv_ | sylv | *Rhodnius ecuadoriensis* | Bramaderos | Paltas | -4,076 | -79,809 | TcI (*T. cruzi*) |
| 54 | TBR249 | *LOJA*_Sylv_ | sylv | *Rhodnius ecuadoriensis* | Bramaderos | Paltas | -4,079 | -79,807 | TcI (*T. cruzi*) |
| 55 | TBR248 | *LOJA*_Sylv_ | sylv | *Rhodnius ecuadoriensis* | Bramaderos | Paltas | -4,079 | -79,807 | TcI (*T. cruzi*) |
| 56 | TBR247 | *LOJA*_Sylv_ | sylv | *Rhodnius ecuadoriensis* | Bramaderos | Paltas | -4,079 | -79,807 | TcI (*T. cruzi*) |
| 57 | TBR136 | *LOJA*_Sylv_ | peri | *Rhodnius ecuadoriensis* | Bramaderos | Paltas | -4,072 | -79,834 | TcI (*T. cruzi*) |
| 58 | TST216 | *LOJA*_Sylv_ | dom | *Panstrongylus chinai* | Santa Ester | Gonzanamá | -4,125 | -79,558 | TcI (*T. cruzi*) |
| 59 | TBR152 | *LOJA*_Sylv_ | sylv | *Rhodnius ecuadoriensis* | Bramaderos | Paltas | -4,076 | -79,809 | TcI (*T. cruzi*) |
| 60 | CG605 | *LOJA*_Sylv_ | sylv | *Rhodnius ecuadoriensis* | La Ciénega | Celica | -4,241 | -79,583 | TcI (*T. cruzi*) |
| 61 | TGN125 | *LOJA*_Sylv_ | sylv | *Rhodnius ecuadoriensis* | El Guineo | Celica | -4,195 | -80,035 | TcI (*T. cruzi*) |
| 62 | CG1031 | *LOJA*_Sylv_ | sylv | *Rhodnius ecuadoriensis* | La Ciénega | Celica | -4,213 | -80,103 | TcI (*T. cruzi*) |
| 63 | TEX223 | *LOJA*_Sylv_ | sylv | *Rhodnius ecuadoriensis* | La Extensa | Catamayo | -4,044 | -79,36 | TcI (*T. cruzi*) |
| 64 | JQ803 | *LOJA*_Sylv_ | sylv | *Rhodnius ecuadoriensis* | Jacapo | Quilanga | -4,364 | -79,416 | TcI (*T. cruzi*) |
| 65 | TGL031 | *LOJA*_Sylv_ | sylv | *Rhodnius ecuadoriensis* | Galápagos | Quilanga | -4,353 | -79,432 | TcI (*T. cruzi*) |
| 66 | TGL032 | *LOJA*_Sylv_ | sylv | *Rhodnius ecuadoriensis* | Galápagos | Quilanga | -4,353 | -79,432 | TcI (*T. cruzi*) |
| 67 | GL500 | *LOJA*_Sylv_ | sylv | *Rhodnius ecuadoriensis* | Galápagos | Quilanga | -4,352 | -79,434 | TcI (*T. cruzi*) |
| 68 | TJU095 | *LOJA*_Sylv_ | dom | *Triatoma carrioni* | Jurupe | Gonzanamá | -4,184 | -79,521 | TcI (*T. cruzi*) |
| 69 | TGL041 | *LOJA*_Sylv_ | sylv | *Rhodnius ecuadoriensis* | Galápagos | Quilanga | -4,353 | -79,432 | TcI (*T. cruzi*) |
| 70 | TGL049 | *LOJA*_Sylv_ | sylv | *Rhodnius ecuadoriensis* | Galápagos | Quilanga | -4,348 | -79,432 | TcI (*T. cruzi* / *T. rangeli*) |
| 71 | TBR209 | *LOJA*_Sylv_ | sylv | *Rhodnius ecuadoriensis* | Bramaderos | Paltas | -4,076 | -79,809 | TcI (*T. cruzi* / *T. rangeli*) |
| 72 | TBR241 | *LOJA*_Sylv_ | sylv | *Rhodnius ecuadoriensis* | Bramaderos | Paltas | -4,076 | -79,809 | TcI (*T. cruzi* / *T. rangeli*) |
| 73 | TBR200 | *LOJA*_Sylv_ | sylv | *Rhodnius ecuadoriensis* | Bramaderos | Paltas | -4,077 | -79,808 | TcI (*T. cruzi*) |
| 74 | TBR204 | *LOJA*_Sylv_ | sylv | *Rhodnius ecuadoriensis* | Bramaderos | Paltas | -4,077 | -79,808 | TcI (*T. cruzi*) |
| 75 | TBR154 | *LOJA*_Sylv_ | sylv | *Rhodnius ecuadoriensis* | Bramaderos | Paltas | -4,076 | -79,809 | TcI (*T. cruzi*) |
| 76 | TBR194 | *LOJA*_Sylv_ | sylv | *Rhodnius ecuadoriensis* | Bramaderos | Paltas | -4,079 | -79,825 | TcI (*T. cruzi*) |
| 77 | BR1349 | *LOJA*_Sylv_ | sylv | *Sciurius stramineus (squirrel)* | Bramaderos | Paltas | -4,078 | -79,806 | TcI (*T. cruzi*) |
| 78 | CE702 | *LOJA*_Sylv_ | sylv | *Rhodnius ecuadoriensis* | Coamine | Paltas | -4,125 | -79,623 | TcI (*T. cruzi*) |
| 79 | CE704 | *LOJA*_Sylv_ | sylv | *Rhodnius ecuadoriensis* | Coamine | Paltas | -4,125 | -79,623 | TcI (*T. cruzi*) |
| 80 | TBR226 | *LOJA*_Sylv_ | sylv | *Rhodnius ecuadoriensis* | Bramaderos | Paltas | -4,082 | -79,804 | TcI (*T. cruzi* / *T. rangeli*) |
| 81 | TBR112 | *LOJA*_Sylv_ | dom | *Panstrongylus chinai* | Bramaderos | Paltas | -4,083 | -79,829 | TcI (*T. cruzi*) |

**^§^** decimal degrees

dom: domestic

peri: peridomestic

sylv: sylvatic
