# Supplementary material for: Sex, Subdivision, and Domestic Dispersal of Trypanosoma cruzi Lineage I in Southern Ecuador
Source: PLoS Negl Trop Dis. 2010 Dec 14;4(12):e915. doi: 10.1371/journal.pntd.0000915 (PMC3001902; doi:10.1371/journal.pntd.0000915)
Supplement: Table S2 — Trypanosoma cruzi primers employed in this study. (0.04 MB DOC) [file pntd.0000915.s003.doc]

**Table S2. *Trypanosoma cruzi* primers employed in this study**

| **Chromosome^§§^**  **(fluorescent dye)** | **Position^¶^** | **Primer code^¶¶^** | **Forward/Reverse Primer (5'-3')** |
| --- | --- | --- | --- |
| 39-S | 171.499 | 6925(TG)b | GAAACGCACTCACCCACAC |
| (nedp8a) |  |  | GGTAGCAACGCCAAACTTTC |
| 27-S | - | 10101(TAA)a | CCGCGGTAGAAGAACCATAA |
| (vicp1b) |  |  | TGCGTATTCACGACGAGAAG |
| 19-S | - | TcUn3 | CTTAAAGAGATACAAGAGGGAAGG |
| (petp4a) |  |  | CTGTTATTTCAATAACACGGGG |
| 39-S | 212.18 | 6925(CT) | CATCAAGGAAAAACGGAGGA |
| (vicp2a) |  |  | CGGTACCACCTCAAGGAAAG |
| 10-P | - | 6855(TA)(GA) | TGTGATCAACGCGCATAAAT |
| (ITETP12a) |  |  | TTCCATTGCCTCGTTTTAGA |
| 6-S | - | mclf10 | GCGTAGCGATTCATTTCC |
| (famp1b) |  |  | ATCCGCTACCACTATCCAC |
| 37-P | - | 10187(TA) | AGAAAAAGGTTTACAACGAGCG |
| (ITETP4a) |  |  | CGATGGAGAACGTGAAACAA |
| 40-P | 613.8 | 11283(TA)b | AACATCCTCCACCTCACAGG |
| (fam8b) |  |  | TTTGAATGCGAGGTGGTACA |
| 40-P | 542.157 | 11283(TCG) | ACCACCAGGAGGACATGAAG |
| (famp8a) |  |  | TGTACACGGAACAGCGAAG |
| 39-S | 49.862 | 7093(TA)c | CGTGTGCACAGGAGAGAAAA |
| (tetp8a) |  |  | CGTTTGGAGGAGGATTGAGA |

**^§§^** Weatherly et al. (27)

**^¶^** Relative map distances are given for multiple loci on the same chromosome.

**^¶¶^** Llewellyn et al. (16)
