# Supplementary material for: Sex, Subdivision, and Domestic Dispersal of Trypanosoma cruzi Lineage I in Southern Ecuador
Source: PLoS Negl Trop Dis. 2010 Dec 14;4(12):e915. doi: 10.1371/journal.pntd.0000915 (PMC3001902; doi:10.1371/journal.pntd.0000915)
Supplement: Table S3 — Allele sizes (in base pairs) from 10 loci analyzed (fluorescent dye) from 81 T. cruzi isolates from Loja Province, Ecuador. (0.30 MB DOC) [file pntd.0000915.s004.doc]

**Table S3.** Allele sizes (in base pairs) from 10 loci analyzed (fluorescent dye) from 81 *T. cruzi* isolates from Loja Province, Ecuador.

|  | **Sample code** | **39-S (nedp8a)** | **27-S (vicp1b)** | **19-S (petp4a)** | **39-S (vicp2a)** | **10-P (1tetp12a)** | **6-S (famp1b)** | **37-P (1tetp4a)** | **40-P (fam8b)** | **40-P (famp8a)** | **39-S (tetp8a)** |
| --- | --- | --- | --- | --- | --- | --- | --- | --- | --- | --- | --- |
| 1 | TJU034 | 109 | 154 | 154 | 176 | 151 | 187 | 114 | 177 | 128 | 191 |
|  |  | 109 | 156 | 162 | 176 | 151 | 189 | 114 | 179 | 132 | 205 |
| 2 | TJU030 | 109 | 154 | 154 | 176 | 151 | 187 | 114 | 177 | 128 | 191 |
|  |  | 109 | 156 | 160 | 176 | 151 | 189 | 114 | 179 | 132 | 205 |
|  |  |  |  | 162 |  |  |  |  |  |  |  |
| 3 | BR6383 | 109 | 154 | 156 | 176 | 151 | 187 | 116 | 177 | 128 | 191 |
|  |  | 109 | 154 | 160 | 176 | 151 | 189 | 116 | 179 | 132 | 191 |
| 4 | TBR123 | 109 | 154 | 154 | 176 | 151 | 189 | 114 | 177 | 132 | 191 |
|  |  | 109 | 154 | 156 | 176 | 151 | 189 | 116 | 177 | 132 | 191 |
|  |  |  |  | 162 |  |  |  |  |  |  |  |
| 5 | GA817 | 109 | 154 | 162 | 174 | 151 | 189 | 116 | 177 | 132 | 191 |
|  |  | 109 | 156 | 162 | 176 | 151 | 189 | 116 | 179 | 132 | 191 |
| 6 | BR1331 | 109 | 154 | 152 | 174 | 151 | 189 | 114 | 177 | 128 | 191 |
|  |  | 109 | 166 | 160 | 176 | 151 | 189 | 116 | 179 | 132 | 205 |
| 7 | CE682 | 109 | 162 | 152 | 174 | 151 | 189 | 114 | 177 | 128 | 191 |
|  |  | 109 | 166 | 160 | 176 | 151 | 189 | 114 | 179 | 128 | 191 |
| 8 | SJ778 | 109 | 154 | 152 | 174 | 151 | 179 | 114 | 177 | 128 | 191 |
|  |  | 109 | 166 | 164 | 176 | 151 | 187 | 114 | 177 | 132 | 191 |
|  |  |  | 174 |  |  |  | 191 |  |  | 136 |  |
| 9 | SJ1147 | 109 | 166 | 152 | 174 | 151 | 187 | 114 | 177 | 128 | 191 |
|  |  | 109 | 166 | 152 | 176 | 151 | 189 | 114 | 177 | 132 | 191 |
| 10 | RT672 | 109 | 154 | 152 | 176 | 151 | 187 | 114 | 177 | 132 | 191 |
|  |  | 109 | 166 | 156 | 176 | 151 | 189 | 114 | 177 | 132 | 191 |
|  |  |  |  |  |  |  | 191 |  |  |  |  |
| 11 | SJ713 | 109 | 154 | 152 | 174 | 151 | 191 | 114 | 177 | 128 | 191 |
|  |  | 109 | 166 | 156 | 176 | 151 | 191 | 114 | 177 | 132 | 191 |
| 12 | CG1232 | 109 | 154 | 156 | 174 | 151 | 191 | 114 | 177 | 132 | 189 |
|  |  | 109 | 154 | 160 | 176 | 151 | 191 | 114 | 177 | 132 | 191 |
| 13 | CE1126 | 109 | 166 | 152 | 174 | 151 | 179 | 114 | 177 | 128 | 205 |
|  |  | 109 | 166 | 162 | 176 | 151 | 187 | 114 | 179 | 132 | 205 |
| 14 | TGL004 | 109 | 154 | 156 | 174 | 151 | 187 | 116 | 177 | 132 | 191 |
|  |  | 109 | 162 | 162 | 174 | 151 | 189 | 116 | 177 | 132 | 205 |
| 15 | CQ800 | 109 | 154 | 152 | 174 | 151 | 179 | 116 | 175 | 132 | 191 |
|  |  | 109 | 156 | 154 | 176 | 151 | 189 | 116 | 179 | 132 | 191 |
|  |  |  | 162 | 156 |  |  |  |  |  |  |  |
|  |  |  | 166 |  |  |  |  |  |  |  |  |
| 16 | SJ1083 | 109 | 154 | 152 | 174 | 151 | 179 | 110 | 177 | 128 | 191 |
|  |  | 109 | 162 | 156 | 176 | 151 | 191 | 114 | 181 | 132 | 191 |
|  |  |  | 166 | 162 |  |  |  |  |  | 136 |  |
| 17 | BR1329 | 106 | 154 | 152 | 172 | 151 | 179 | 114 | 173 | 132 | 189 |
|  |  | 109 | 162 | 160 | 176 | 151 | 189 | 114 | 177 | 136 | 191 |
| 18 | TBR199 | 106 | 154 | 152 | 172 | 151 | 179 | 110 | 173 | 132 | 191 |
|  |  | 109 | 162 | 154 | 174 | 151 | 179 | 114 | 177 | 136 | 191 |
|  |  |  |  | 162 | 176 |  |  | 116 |  |  |  |
| 19 | TGN081 | 106 | 154 | 152 | 174 | 149 | 179 | 114 | 175 | 136 | 189 |
|  |  | 109 | 162 | 162 | 176 | 151 | 179 | 114 | 175 | 136 | 203 |
| 20 | TBR193 | 106 | 162 | 152 | 172 | 149 | 179 | 110 | 173 | 136 | 189 |
|  |  | 109 | 162 | 154 | 174 | 149 | 179 | 110 | 175 | 136 | 189 |
|  |  |  |  | 162 |  |  |  |  |  |  |  |
| 21 | TBR163 | 109 | 162 | 152 | 174 | 149 | 179 | 110 | 173 | 136 | 189 |
|  |  | 109 | 162 | 152 | 176 | 149 | 179 | 110 | 175 | 136 | 191 |
|  |  |  |  |  |  |  |  |  |  |  | 197 |
| 22 | TBR230 | 106 | 162 | 152 | 174 | 149 | 179 | 110 | 173 | 136 | 187 |
|  |  | 109 | 162 | 152 | 176 | 149 | 179 | 114 | 175 | 136 | 189 |
|  |  |  |  |  |  |  |  |  | 179 |  |  |
| 23 | TBR237 | 106 | 162 | 152 | 174 | 149 | 179 | 110 | 173 | 136 | 191 |
|  |  | 109 | 162 | 152 | 176 | 151 | 179 | 110 | 175 | 136 | 193 |
| 24 | TBR153 | 106 | 162 | 152 | 174 | 151 | 179 | 110 | 173 | 136 | 191 |
|  |  | 109 | 162 | 152 | 176 | 151 | 179 | 110 | 175 | 136 | 193 |
| 25 | TBR220 | 106 | 162 | 152 | 174 | 151 | 179 | 110 | 173 | 136 | 189 |
|  |  | 109 | 162 | 162 | 176 | 151 | 179 | 110 | 175 | 136 | 191 |
| 26 | TBR218 | 106 | 162 | 152 | 174 | ND | 179 | 110 | 173 | 136 | 189 |
|  |  | 109 | 164 | 152 | 176 | ND | 179 | 110 | 175 | 136 | 191 |
| 27 | EX320 | 106 | 162 | 152 | 172 | 147 | 179 | 110 | 175 | 136 | 187 |
|  |  | 109 | 162 | 152 | 176 | 149 | 179 | 110 | 175 | 136 | 189 |
| 28 | EX319 | 106 | 162 | 152 | 172 | 147 | 179 | 110 | 175 | 136 | 187 |
|  |  | 109 | 162 | 152 | 176 | 147 | 179 | 110 | 175 | 136 | 189 |
| 29 | TSA170 | 106 | 162 | 152 | 174 | 151 | 179 | 108 | 175 | 136 | 187 |
|  |  | 109 | 162 | 152 | 176 | 151 | 179 | 110 | 179 | 136 | 189 |
| 30 | SJ712 | 109 | 162 | 152 | 174 | 151 | 179 | 110 | 175 | 136 | 187 |
|  |  | 109 | 166 | 152 | 174 | 151 | 179 | 110 | 181 | 136 | 187 |
| 31 | CE1141 | 109 | 162 | 152 | 174 | 151 | 179 | 110 | 175 | 136 | 187 |
|  |  | 109 | 166 | 152 | 174 | 151 | 179 | 110 | 181 | 136 | 187 |
| 32 | SJ1079 | 109 | 162 | 152 | 174 | 151 | 179 | 110 | 175 | 136 | 187 |
|  |  | 109 | 166 | 152 | 174 | 151 | 179 | 110 | 181 | 136 | 187 |
| 33 | SJ1080 | 109 | 162 | 152 | 174 | 151 | 179 | 110 | 175 | 136 | 187 |
|  |  | 109 | 166 | 152 | 174 | 151 | 179 | 110 | 181 | 136 | 187 |
| 34 | SJ744 | 109 | 162 | 152 | 174 | 151 | 179 | 110 | 175 | 118 | 187 |
|  |  | 109 | 166 | 152 | 174 | 151 | 179 | 110 | 181 | 136 | 187 |
| 35 | SJ795 | 109 | 162 | 152 | 174 | 151 | 179 | 110 | 175 | 118 | 187 |
|  |  | 109 | 166 | 152 | 174 | 151 | 179 | 110 | 181 | 136 | 187 |
| 36 | SJ1081 | 109 | 162 | 152 | 174 | 151 | 179 | 110 | 175 | 136 | 187 |
|  |  | 109 | 166 | 162 | 174 | 151 | 179 | 110 | 181 | 136 | 187 |
| 37 | SJ1127 | 109 | 162 | 154 | 174 | 151 | 179 | 110 | 175 | 136 | 187 |
|  |  | 109 | 166 | 162 | 174 | 151 | 179 | 110 | 181 | 136 | 187 |
| 38 | SJ737 | 106 | 166 | 152 | 172 | 151 | 179 | 110 | 175 | 136 | 187 |
|  |  | 109 | 166 | 152 | 174 | 151 | 179 | 110 | 181 | 136 | 197 |
| 39 | SJ718 | 109 | 162 | 152 | 172 | 151 | 179 | 110 | 175 | 136 | 187 |
|  |  | 109 | 166 | 152 | 172 | 151 | 179 | 110 | 181 | 136 | 197 |
| 40 | SJ721 | 109 | 162 | 152 | 172 | 151 | 179 | 110 | 175 | 136 | 187 |
|  |  | 109 | 166 | 152 | 172 | 151 | 179 | 110 | 181 | 136 | 197 |
| 41 | SJ717 | 109 | 162 | 152 | 172 | 151 | 179 | 110 | 175 | 136 | 187 |
|  |  | 109 | 166 | 152 | 172 | 151 | 179 | 110 | 181 | 136 | 197 |
| 42 | SJ796 | 106 | 162 | 152 | 172 | 151 | 179 | 110 | 175 | 136 | 187 |
|  |  | 109 | 166 | 152 | 174 | 151 | 179 | 110 | 181 | 136 | 187 |
| 43 | SJ723 | 106 | 162 | 152 | 174 | 151 | 179 | 110 | 175 | 136 | 187 |
|  |  | 109 | 166 | 152 | 174 | 151 | 179 | 110 | 181 | 136 | 189 |
| 44 | SJ1082 | 106 | 162 | 152 | 172 | 151 | 179 | 110 | 175 | 136 | 187 |
|  |  | 106 | 166 | 162 | 174 | 151 | 179 | 110 | 181 | 136 | 189 |
| 45 | SJ1162 | 106 | 162 | 152 | 172 | 151 | 179 | 110 | 175 | 136 | 187 |
|  |  | 106 | 166 | 152 | 174 | 151 | 179 | 110 | 181 | 136 | 189 |
| 46 | TBR224 | 106 | 162 | 152 | 174 | 149 | 179 | 110 | 175 | 136 | 189 |
|  |  | 109 | 166 | 152 | 174 | 151 | 179 | 110 | 175 | 136 | 189 |
| 47 | TBR238 | 106 | 162 | 152 | 174 | 149 | 179 | 110 | 175 | 136 | 189 |
|  |  | 109 | 166 | 152 | 174 | 151 | 179 | 110 | 175 | 136 | 189 |
| 48 | SJ738 | 106 | 162 | 152 | 174 | 151 | 179 | 110 | 175 | 136 | 187 |
|  |  | 109 | 166 | 152 | 174 | 151 | 179 | 110 | 175 | 136 | 189 |
|  |  |  |  |  |  |  |  |  |  |  | 197 |
| 49 | TBR150 | 106 | 162 | 152 | 174 | 151 | 179 | 110 | 175 | 136 | 187 |
|  |  | 106 | 166 | 152 | 174 | 151 | 179 | 114 | 175 | 136 | 187 |
|  |  |  | 174 |  |  |  |  |  |  |  |  |
| 50 | TBR155 | 106 | 166 | 152 | 174 | 149 | 179 | 110 | 175 | 118 | 187 |
|  |  | 106 | 174 | 152 | 174 | 151 | 179 | 114 | 175 | 136 | 187 |
| 51 | TBR149 | 106 | 166 | 152 | 174 | 149 | 179 | 110 | 175 | 136 | 187 |
|  |  | 106 | 174 | 160 | 174 | 151 | 179 | 114 | 175 | 136 | 187 |
| 52 | TBR215 | 106 | 162 | 152 | 174 | 151 | 179 | 108 | 175 | 118 | 187 |
|  |  | 106 | 166 | 156 | 174 | 151 | 179 | 108 | 175 | 136 | 187 |
| 53 | TBR240 | 106 | 162 | 152 | 174 | 147 | 179 | 110 | 175 | 136 | 187 |
|  |  | 109 | 166 | 158 | 174 | 151 | 179 | 114 | 175 | 136 | 187 |
|  |  |  |  | 162 |  |  |  |  |  |  |  |
| 54 | TBR249 | 106 | 162 | 152 | 174 | 147 | 179 | 110 | 175 | 136 | 187 |
|  |  | 109 | 166 | 158 | 174 | 147 | 179 | 110 | 175 | 136 | 197 |
| 55 | TBR248 | 106 | 162 | 152 | 174 | 147 | 179 | 110 | 175 | 136 | 187 |
|  |  | 109 | 166 | 158 | 174 | 147 | 179 | 110 | 175 | 136 | 197 |
| 56 | TBR247 | 106 | 162 | 152 | 174 | 149 | 179 | 106 | 175 | 136 | 187 |
|  |  | 109 | 166 | 158 | 174 | 149 | 179 | 106 | 175 | 136 | 187 |
| 57 | TBR136 | 106 | 162 | 152 | 174 | 151 | 179 | 110 | 173 | 136 | 187 |
|  |  | 109 | 164 | 152 | 174 | 151 | 179 | 114 | 173 | 136 | 187 |
| 58 | TST216 | 109 | 164 | 152 | 174 | 149 | 179 | 114 | 175 | 136 | 187 |
|  |  | 109 | 164 | 152 | 174 | 149 | 179 | 114 | 175 | 136 | 195 |
| 59 | TBR152 | 109 | 162 | 152 | 174 | 151 | 179 | 110 | 175 | 136 | 191 |
|  |  | 109 | 174 | 152 | 174 | 153 | 179 | 110 | 175 | 136 | 191 |
| 60 | CG605 | 106 | 162 | 152 | 174 | 141 | 179 | 110 | 175 | 122 | 189 |
|  |  | 106 | 164 | 154 | 176 | 149 | 179 | 110 | 175 | 136 | 195 |
| 61 | TGN125 | 106 | 160 | 152 | 172 | 141 | 179 | 110 | 175 | 118 | 189 |
|  |  | 106 | 164 | 152 | 174 | 149 | 179 | 110 | 177 | 136 | 193 |
| 62 | CG1031 | 106 | 164 | 152 | 172 | 147 | 179 | 106 | 175 | 132 | 187 |
|  |  | 106 | 172 | 152 | 176 | 147 | 179 | 106 | 175 | 136 | 195 |
| 63 | TEX223 | 106 | 160 | 152 | 172 | 149 | 179 | 110 | 175 | 118 | 189 |
|  |  | 109 | 166 | 152 | 176 | 149 | 179 | 110 | 181 | 136 | 189 |
| 64 | JQ803 | 106 | 160 | 152 | 174 | 149 | 179 | 102 | 173 | 118 | 195 |
|  |  | 106 | 166 | 152 | 174 | 149 | 179 | 102 | 175 | 136 | 195 |
| 65 | TGL031 | 106 | 160 | 152 | 172 | 151 | 179 | 106 | 175 | 136 | 189 |
|  |  | 109 | 168 | 152 | 174 | 151 | 179 | 106 | 175 | 136 | 195 |
| 66 | TGL032 | 106 | 160 | 152 | 172 | ND | 179 | 106 | 175 | 136 | 189 |
|  |  | 109 | 168 | 152 | 174 | ND | 179 | 106 | 175 | 136 | 195 |
| 67 | GL500 | 106 | 160 | 152 | 172 | 151 | 179 | 106 | 175 | 136 | 189 |
|  |  | 109 | 168 | 152 | 174 | 151 | 179 | 106 | 175 | 136 | 195 |
| 68 | TJU095 | 106 | 160 | 152 | 172 | 151 | 179 | 106 | 175 | 136 | 189 |
|  |  | 109 | 168 | 162 | 174 | 151 | 179 | 106 | 175 | 136 | 195 |
| 69 | TGL041 | 106 | 160 | 152 | 172 | 151 | 179 | 106 | 175 | 136 | 189 |
|  |  | 109 | 168 | 152 | 174 | 151 | 179 | 106 | 175 | 136 | 189 |
| 70 | TGL049 | 106 | 160 | 152 | 172 | 151 | 179 | ND | 175 | 136 | 189 |
|  |  | 109 | 168 | 152 | 174 | 151 | 179 | ND | 175 | 136 | 195 |
| 71 | TBR209 | 106 | 162 | 152 | 174 | 151 | 179 | 114 | 173 | 136 | 187 |
|  |  | 106 | 166 | 152 | 174 | 151 | 179 | 114 | 179 | 136 | 197 |
| 72 | TBR241 | 106 | 162 | 152 | 172 | 151 | 179 | 110 | 173 | 136 | 187 |
|  |  | 106 | 166 | 152 | 174 | 151 | 179 | 114 | 179 | 136 | 189 |
|  |  |  | 174 |  |  |  |  |  |  |  | 191 |
| 73 | TBR200 | 106 | 162 | 152 | 172 | ND | 179 | 110 | 173 | 136 | 189 |
|  |  | 106 | 162 | 152 | 174 | ND | 179 | 110 | 179 | 136 | 191 |
| 74 | TBR204 | 106 | 162 | 152 | 172 | 151 | 179 | 110 | 173 | 136 | 189 |
|  |  | 106 | 162 | 152 | 174 | 151 | 179 | 110 | 179 | 136 | 191 |
| 75 | TBR154 | 106 | 162 | 152 | 172 | 147 | 179 | 106 | 177 | 136 | 189 |
|  |  | 106 | 166 | 152 | 174 | 147 | 179 | 110 | 177 | 136 | 191 |
| 76 | TBR194 | 106 | 162 | 152 | 174 | 147 | 179 | 108 | 173 | 136 | 189 |
|  |  | 106 | 174 | 154 | 174 | 147 | 179 | 110 | 173 | 136 | 189 |
| 77 | BR1349 | 106 | 162 | 152 | 174 | 147 | 179 | 108 | 173 | 132 | 191 |
|  |  | 106 | 174 | 154 | 174 | 147 | 179 | 110 | 173 | 132 | 191 |
|  |  |  |  | 162 |  |  |  |  |  |  |  |
| 78 | CE702 | 106 | 162 | 152 | 172 | 149 | 179 | 108 | 175 | 136 | 189 |
|  |  | 106 | 164 | 162 | 174 | 149 | 179 | 108 | 177 | 136 | 189 |
| 79 | CE704 | 106 | 162 | 152 | 172 | ND | 179 | 108 | 175 | 136 | 189 |
|  |  | 106 | 164 | 152 | 174 | ND | 179 | 108 | 177 | 136 | 189 |
| 80 | TBR226 | 106 | 163 | 152 | 172 | 147 | 179 | 108 | 173 | 136 | 189 |
|  |  | 106 | 174 | 152 | 174 | 147 | 179 | 108 | 175 | 136 | 189 |
| 81 | TBR112 | 103 | 162 | 152 | 174 | 151 | 179 | 108 | 177 | 136 | 187 |
|  |  | 106 | 164 | 158 | 176 | 151 | 179 | 108 | 177 | 136 | 189 |
|  |  | 109 |  | 162 |  |  |  |  |  |  | 191 |
| ND: no data | | | | | | | | | | | |
